# Health Risks Faced by Turkish Agricultural Workers

**DOI:** 10.1155/2014/185342

**Published:** 2014-06-25

**Authors:** Hülya Çakmur

**Affiliations:** Department of Family Medicine, School of Medicine, University of Kafkas, 36100 Kars, Turkey

## Abstract

*Background*. Individuals who make a living through agriculture and animal husbandry are faced with a variety of physical and psychological health risks. In many international studies, it has been shown that these risks can result in disease. The purpose of this study is to summarize the health risks faced by Turkish agricultural workers. *Materials and Methods*. This study used a nonrandom, convenience sample. The biopsychosocial health statuses of 177 farmers from 11 central villages in Kars, Turkey, were examined.* Results*. It was determined that the depression rate among the study group was 62.1%, the rate of physical health problems was 52.0%, and the rate of social isolation was 53.7%. There was a statistically significant difference between the depression scale scores and lower education levels, having ≥ three children, and physical health problems, as well as the physical condition of the farmers' homes. There was a significant difference between poor physical health and older age, lower education levels, having ≥ three children, and social isolation.* Conclusions*. In providing data-based evidence, it is believed that this study will contribute considerably to understanding the causality of health problems in this population and in planning the development of public health and veterinary services based on regional needs.

## 1. Introduction

Eastern Anatolia is the region of Turkey in which agriculture and animal husbandry are most extensive. In the Northeastern Anatolia Region, which encompasses the Kars Province, agriculture and animal husbandry comprised 24.6% of the gross domestic product (GDP) in 2009; the national average was 8.5% [1]. In the Kars Province, 56.95% of the population lives in rural areas; the average percentage of the population living in rural areas is 22.72% nationwide [2]. Agriculture and animal husbandry are suited to Kars Province because of its geographic and climatic features and large rural population. However, in the last thirty-five years, Turkey has lost the characteristics of an agricultural country [3]. The Turkish Institute of Statistics (TÜİK) has not performed a census of the number of individuals working in agriculture since 2001 [4].

Nevertheless, residents in rural areas still rely on agriculture and animal husbandry for their livelihoods, mostly without any state support. The Eastern Anatolia Region ranks below the nationwide average for all socioeconomic criteria [5]. The agricultural, industrial, and service sectors in the Eastern Anatolia Region are all underdeveloped, while other social development indicators for the region, such as population, health, education, and prosperity, also rank below national averages [5]. No studies have specifically evaluated the health statuses of farmers living in undeveloped regions of Turkey. However, the high population movement rates (i.e., emigration) and infant mortality in the region are important indicators of the lack of socioeconomic development. In the Northeastern Anatolia Region, the infant mortality rate is 12.8, which is above the national average of 10.1 [6]. As Wagstaff stated, “poverty” and illness are intertwined; poor countries tend to have worse health outcomes than better-off countries [7]. In Turkey, poverty is severe in rural areas, such as Eastern Anatolia. The absolute poverty line for Turkey was US $4 per capita per day (2006). Saatçi and Akpinar had the highest poverty rates for agricultural workers (46.6%), and in Eastern and Southeastern Anatolia, 65.6% of the people working in the agricultural sector were poor [3]. Dansuk has shown that poverty is directly correlated with low education levels in Turkey. He stated that in the general population, 97.9% of poor people were illiterate, had rudimentary reading and writing skills, and had graduated from primary school [8]. Koruk et al. showed that farm workers' health service utilization is limited by their migrant lifestyles and the limited number of healthcare facilities in Turkey's agricultural region [9].

It has been previously demonstrated that the rate of occupational injury and death in farming is greater than those observed in other occupations [10, 11]. Muscle and bone injuries caused by carrying, lifting, and pulling heavy objects, as well as chronic musculoskeletal pain, are frequently reported in agriculture and animal husbandry workers [12, 13]. In the United States, a significant relationship was demonstrated between the incidence of lung and lymphohematopoietic cancers and raising poultry and cattle [14]. It has also been reported that compared to individuals in other occupations, individuals working in agriculture are more predisposed to developing occupational diseases, such as asthma and respiratory ailments caused by chemicals, dust, and allergens [15].

Because of the high rates of occupation-related diseases, accidents, and injuries, farming is considered to be the second most hazardous occupation worldwide, after mining [16]. Common health problems experienced by farmers include dermatoses, adverse effects associated with exposure to excessively hot and cold conditions, zoonoses, hearing loss, and certain cancers [17, 18]. Garcia and McCarthy stated that “positive health” has three linked dimensions (social, psychological, and physical) that should all be included when measuring health [19]. The aim of the current study was to examine the biological, psychological, and social health of farmers in Kars Province, which is characterized by a relatively low level of socioeconomic development. In this context, this study also attempted to determine the factors that might affect the health of farmers in the region.

## 2. Materials and Methods

This cross-sectional survey, which was conducted from March to May 2013, examined 177 subjects subsisting on agriculture and animal husbandry in 11 different villages in Kars Province. The Kafkas University Medical Faculty Ethics Committee (protocol number 050-99/13) approved the study, and all participants provided verbal informed consent. Data were collected through face-to-face interviews.

The sample size was calculated using the Epi Info Statcalc program. In this nonrandom, convenience sample, the dependent variable was bio-psycho-social health. The independent variables were age, gender, marital status, education, socioeconomic status, agricultural pesticide and fertilizer uses, agricultural machinery use, number of siblings, government support for agriculture, number of cattle, smoking, and alcohol consumption.

A 38-item questionnaire consisting of open- and closed-ended questions to assess the social and physical health of the subjects was used as a data collection tool. The survey questions were prepared using the Copenhagen criteria (i.e., social, psychological, and physical health measures) to evaluate the health statuses of the participants [19].

To evaluate social health, the questionnaire included questions assessing social integration, overall life satisfaction, trust issues, perception of safety, solidarity and sharing of tasks between individuals, use of common machines and tools, sense of belonging and socioeconomic security among individuals, and individual thoughts regarding the ability to earn a living (even if they were not currently working) [19].

In addition to monthly income, the economic statuses of the subjects were evaluated using independent questions to assess the land and number of animals owned, physical conditions of their houses (i.e., the number of rooms and presence of electricity and running water), agricultural machines owned (both vehicles and tools), and government support received.

Statistical evaluations for economic status were based on the farmers' average monthly incomes. The physical health statuses of the farmers were evaluated by examining the available medical records for any information regarding acute health problems, past accidents, chronic diseases, medication use, and occupational diseases. Temporary health problems that did not affect their daily activities were not considered [19].

Smoking and alcohol consumption were assessed using closed-ended questions; however, the duration and quantity of use were not evaluated. The mental health statuses of the participants were assessed only for depression using the Beck Depression Scale (BDS). Other, relatively uncommon, mental health disorders (e.g., schizophrenia, bipolar disorder, and other mental health disorders) were excluded from this study.

The BDS consists of 21 items that were scored between 0 and 3 points to evaluate the presence and severity of depression. Nine of these items are part of the depression subscale; six items are part of the neurotic subscale, and six items are part of the somatic subscale [19, 20]. The lowest possible score for this scale is 0, and the highest possible score is 63. A score of 14 points indicates the presence of clinically relevant depression. Individuals with a Beck score ≥ 9 were considered to be depressed, while individuals with a score < 9 were not considered to be depressed. Validity and reliability analyses were previously performed for this scale. This self-assessment scale does not require the person administering it to undergo any particular training [20].

SPSS software was used for the data analysis. Percentage distributions, frequencies, arithmetic means, and standard deviations (SD) were examined as descriptive statistics. Pearson's chi-squared and Fisher's exact tests were used to compare variables. The threshold for statistical significance was set at *P* < 0.05.

## 3. Results

The study group ranged in age from 15 to 85 years old (mean, 37.66 ± 15.01 years, 77% female). The percentage of illiterate subjects was 23.7%, while 11.9% of the subjects were literate without any schooling, and 40.1% had an elementary school education. It was determined that 75.7% of the subjects had not completed an elementary school education, and 55.9% had monthly incomes ≤ 500 Turkish Liras (US $250). Among the study group, 24.3% of the subjects reported that their incomes had increased in the past 10 years, 35.6% reported that their incomes had decreased in the past 10 years, and 40.1% reported that their incomes had not changed in the past 10 years. A total of 44.1% of the subjects were able to subsist/earn a living but did not feel socioeconomically secure in their villages; 5.6% and 15.3% did not have any electricity or running water, respectively, in their homes; 32.3% worked and subsisted on their own lands; 9.6% owned land but did not work on it; 10.7% worked on their own land but were unable to subsist/earn a living; 57.1% did not own land; 32.7% did not own any animals; 32.8% did not own cattle; 91.5% did not own sheep or goats; 46.4% owned > 10 head of cattle; 55.9% used vehicles for agricultural activities and animal husbandry; 21.5% used pesticides; 39.5% used artificial fertilizers; 29.4% owned their own vehicles and tools; and 24.9% received government support (Table 1). A total of 52.5% of the participants had ≥ three children. In this study, it was determined that the age at which the participants most frequently began working was 11 years old (21.3%). In addition, 2.8% of the study subjects were between 15 and 18 years old. The percentage of children involved in agricultural and animal husbandry activities was 42.4%; 96.0% of the children had started school at the expected age (six). It was determined that 62.1% of subjects had depression, 40.6% had physical health problems, and 46.6% had social health problems (Table 1).

The frequency of depression was three times greater among the subjects who were 35 years old or older, and compared with the subjects who were 34 years old or younger, this difference was statistically significant. No statistically significant difference was observed between social health and age, but a significant relationship was identified between physical health and age. No gender difference was identified for the physical health, social health, and BDS scores of the subjects. A negative, linear, and statistically significant relationship was identified between their education levels and depression scale scores. A statistically significant difference was also observed between their education levels and physical, mental, and social health. No significant relationship was identified between the subjects' physical, mental, and social health and their civil statuses. A significant relationship was identified between the subjects' physical and mental health and their number of children. However, no significant relationship was observed between the subjects' social health and their number of children. It was also determined that an increase in the number of children was accompanied by a decrease in physical health and a significant increase in depression scores (*r* = 0.253). A statistically significant difference was observed between the BDS scores and physical health, age, education level, and number of children, as well as the physical condition of the farmers' homes (i.e., the number of rooms and presence of electricity and running water, Table 2). No significant differences were identified between the BDS scores and gender, civil status, monthly income, smoking status, alcohol use, social support, agricultural machinery and pesticide use, sense of belonging, and receipt of government support. Statistically significant differences were identified between social health and physical health and learning and also between the sense of belonging and physical health and learning. No significant difference was identified between social health and age, gender, marital status, number of children, monthly income, smoking status, alcohol use, agricultural machinery and pesticide use, and BDS scores, as well as the physical conditions of the farmers' homes. Similarly, no significant difference was determined between the subjects' social health and the government support they received. A statistically significant difference was observed between the subjects' physical health and their age, level of education, number of children, BDS scores, and social health. No significant difference was identified between physical health and gender, marital status, smoking status, alcohol use, agricultural machinery and pesticide use, and possession of a large number of cattle, as well as the physical condition of the farmers' homes. Among the study group, 11.9% smoked or used alcohol. A significant difference was identified between the subjects' depression scale scores and the number of rooms in their homes. No statistically significant difference was identified between the number of rooms in the farmers' homes and their physical and social health, and no significant difference was identified between their physical health and the use of pesticides and artificial fertilizers or the ownership of >10 head of cattle. No statistically significant difference was identified between the social, mental, and physical health of the participants and their income levels (Table 2).

## 4. Discussion

In this study, it was determined that biological, psychological, and social health problems were relatively common among farmers and that the most important underlying cause of these health problems was their education levels. The strength of this study is that it is the first study conducted in Kars Province. However, one limitation was the nonhomogenous distribution of gender within the study group. This limitation was caused by the fact that most men from these villages worked outside of the province during the long winter months because of the economic and climatic conditions of the region. Turkey ranked 90 out of 186 countries, according to 2013 Human Development Report (Human Development Index, 0.722) [21]. Per capita gross national income (GNI) does not differ greatly between neighboring countries (13.710 in Turkey, 20.511 in Greece, 11.474 in Bulgaria, 5.005 in Georgia, 10.695 in Iran, and 3.557 in Iraq), but the mean number of years of schooling did differ (6.5 years in Turkey, 10.1 years in Greece, 12.1 years in Georgia, 10.6 years in Bulgaria, 7.8 years in Iran, and 5.6 years in Iraq). The life expectancy at birth is 74.2 years in Turkey, 80.0 years in Greece, 73.9 years in Georgia, 73.6 years in Bulgaria, 73.2 years in Iran, and 69.6 years in Iraq. The 2013 infant mortality rate (per 1000) was 14 in Turkey (according to the records of the Health Ministry of Turkey, 10.1), 3 in Greece, 14 in Syria, 31 in Iraq, 22 in Iran, 11 in Bulgaria, and 20 in Georgia. Additionally, the under-five mortality rate was greater in Turkey (18 per 1000) [6, 21]. It has been shown that the population working in the agricultural sector was less compliant with vaccination schedules in rural areas of Turkey [9]. A high rate of subjects in the study group reported that their children also worked in agriculture and animal husbandry (42.4%). Child participation in agricultural and animal husbandry activities is a common observation worldwide. However, it has been reported that a decrease in the working age is also associated with an increased risk of respiratory diseases, musculoskeletal diseases, hearing loss, accidents, and injury [22]. In Canada, it was reported that exposure to agricultural pesticides at an early age was an independent risk factor for developing diabetes among men later in life [23]. Reproductive health problems have also been reported in male children engaged in farming activities [24]. In the United States, the minimum age for working in agriculture and animal husbandry is seventeen. Furthermore, children must also satisfy the criteria described in the North American Guidelines for Children Agricultural Tasks (NAGCAT) before being able to work in agriculture and animal husbandry [25]. Nearly one-third of the individuals working in the agricultural sector in the United States are older than age 65; on a personal level, these individuals define health as the ability to work [26]. In the rural areas of Kars Province, the age at which children generally begin to work has been reported as 11 years, which might have contributed to the high prevalence of health problems observed within the study group. One positive result was that 96.0% of the children started school at the expected age. The number of individuals who work primarily in agriculture and animal husbandry was 8,165,438, according to data from 2001. A large portion (33.4%) of this population was composed of individuals > 50 years old. However, the lowest studied age group (i.e., individuals between 9 and 12 years of age) constituted only 2.9% of this population [2, 4]. In the current study, deterioration in the physical and mental health of the farmers was observed with increasing age, while social health was not affected by age. The worsening of physical health with advancing age is an expected result. The average age in rural areas of Turkey is relatively high and is gradually increasing. While the rural population aged 65 years or older was 1.7 million in 2000 (7.4% of the total rural population), this number increased to 1.9 million in 2010 (to 11.1% of the total rural population). Within the same period, the urban population of individuals who are 65 years of age or older increased from 2.1 million in 2000 (4.7% of the total urban population) to 3.4 million in 2010 (6% of the total urban population). The 2008 results of the Turkish Population and Health Research study provided similar numbers and ratios (an elderly urban population ratio of 5.5% compared to an elderly rural population of 10.1%) [2, 5]. This difference is because of the migration of the younger population to cities while the elderly population remains in rural areas. The migration of retired elderly individuals from urban areas to rural areas has also contributed to this difference. In Sweden, where dairy production and animal husbandry are extensively conducted, research has shown that farmers are more predisposed to diseases, mental health problems, and suicide than other professions. This result is mainly because of their difficult working conditions and their social and environmental responsibilities. It is also because of external factors, such as changing weather conditions, unstable/unpredictable market conditions, and constantly changing state regulations. For this reason, researchers have emphasized the necessity of developing an international program to protect and support farmers and their families, regardless of where they live [27]. It was surprising to note that the current subjects' physical, mental, and social health were not affected by economic factors, such as monthly income or ownership of land, animals, and machinery. A study conducted in rural areas of Canada demonstrated that farmers with a negative perception of their economic status had a higher risk of accidents and injuries [28]. In another study conducted in the United States, low socioeconomic status among farmers was associated with higher rates of disease and injury [29]. In the current study, 55.9% of the subjects perceived themselves as socioeconomically secure in their village despite the fact that they did not work. Approximately half of the participants (40.1%) described their income levels a sun changed over the previous 10 years. I believe that these results reflect the fact that the farmers did not perceive their current economic statuses as sources of problems, likely because they are habituated to their current economic statuses and feel socioeconomically secure in their social environments. The depression levels among the farmers were high. The percentage of female subjects in the study group was 77.4%, and depression is reported more frequently among women. It was unsurprising that an increase in the number of children was associated with deterioration in the mental and physical health of the subjects. However, a study conducted in the United States has demonstrated that the high incidence of depression among female farmers was associated with their exposure to pesticides [30]. There was no statistically significant relationship between pesticide usage and BDS scores in this study. A study in Canada reported that the incidence of suicide was high among male farmers and that men generally avoided asking for help or support for their mental health problems [31]. In a study of farmers affected by droughts in Australia, researchers described mental health as the most important requirement for becoming a healthy individual and observed that individual mental health could be supported by reinforcing their social bonds [32]. Dubos defined health as an individual's “ability to socialize.” The most interesting aspect of the current study was the direct relationship between overall health and the level of education. A negative linear relationship was observed between the farmers' health problems and their education levels. A study in Colombia demonstrated that education led to behavioral changes among farmers, thus allowing them to reduce occupational risks [33]. In Australia, 60% of the land is used for agriculture and animal husbandry; studies there are currently being conducted to develop farmer-oriented educational services and programs. It was reported that providing such education and training to farmers and their families has resulted in a considerable decrease in the incidence of occupational diseases and accidents [34]. Various studies have demonstrated that training sessions to raise the awareness of zoonotic diseases (which are commonly observed among individuals who work in agriculture and raise animals) have contributed significantly to their prevention [35, 36]. No relationship was identified between the farmers' physical, mental, and social health and their low ratio of smoking and alcohol use. This result, which was inconsistent with other results in the literature, was not surprising because women constituted the majority of the study population and there were no questions regarding the quantity and duration of use. This study is the first to measure the bio-psycho-social health of agricultural workers living in an undeveloped area in Turkey. Based on these findings, it is easier to understand the sociodemographic characteristics and health conditions of the farmers in this region. Because of the lack of studies investigating the overall health of agricultural workers in Turkey, these results were not compared with any past results. Inequalities between the Western and Eastern regions of Turkey also affect health statuses. Nevertheless, I believe that if this study was conducted in the Western portion of Turkey, the results would be similar because agricultural workers have low educational levels nationwide. The fact that nearly half of the current study group have not completed their elementary school educations and that nearly one-third were illiterate could account for the high incidence of health problems. Although the distribution of gender within the study group was not homogenous, gender was not identified as a risk factor for the biological, mental, and social health problems. In addition, no relationship was identified between health status and the low economic level of farmers. Although the BDS scores among the participants were not considered to be clinically relevant, this observation requires further study.

## 5. Conclusion

As defined by the World Health Organization, health is a “state of full biological, psychological, and social well-being.” The main purpose of this research was to determine the factors affecting the bio-psycho-social health of agricultural workers living in an undeveloped part of Turkey. This study determined that the most important underlying cause of health problems was the level of education. This result indicates that education is essential to promote health and eradicate poverty.

## Figures and Tables

**Table 1:** Sociodemographic characteristics of participants compared with the Beck scores.

Characteristic	*n*	%	Min	Max	Mean	SD	Beck (*P*)
Age							
≤34	91	51.4	15.00	85.00	37.65	15.019	
≥35	86	48.6					
Gender							
Female	137	77.4					0.491
Male	40	22.6					
Marital status							
Married	124	70.1					0.983
Other	53	29.9					
Number of children							
≤2	84	47.5	0.00	9.00	2.80	2.039	
≥3	93	52.5					
Education level							
Elementary & ↓	134	75.7					
Secondary & ↑	43	24.3					
Physical health							
Good	104	59.4					
Not good	71	40.6					
Economic support							
Receives	89	53.4					0.218
Does not receive	65	46.6					
Beck score							
≤8	67	37.9	0.00	45.00	13.18	10.579	
≥9	110	62.1					
Monthly income							
≤500 TRY	99	55.9	150.00	3000.00	766.10	490.197	0.634
≥501 TRY	78	44.1					
Change in monthly income (in past ten years)							
Increased	43	24.3					0.551
Decreased	63	35.6					
Did not change	71	40.1					
Government support							
Yes	44	24.9					0.814
No	133	75.1					
Number of rooms in house							
≤2	80	45.2	1.00	7.00	2.89	1.234	
≥3	97	54.8					
Land ownership							
Yes	76	42.9					0.095
No	101	57.1					
Number of cattle							
≤9	95	53.7	0.00	60.00	16.40	19.719	0.848
≥10	82	46.3					
Usage of agricultural pesticides							
Yes	38	21.5					0.329
No	139	78.5					
Usage of artificial fertilizers							
Yes	70	39.5					0.479
No	107	60.5					
Usage of work machinery							
Yes	99	55.9					0.870
No	78	44.1					
Child workers							
Yes	75	42.4					0.690
No	102	57.6					
Age when children begin to help							
≤14	49	65.3	2.00	17.00	11.28	4.210	0.975
≥15	26	34.7					
Cooperates only with relatives							
Yes	70	39.5					
No	107	60.5					0.154
Smoking and alcohol use							
Yes	21	11.9					
No	156	88.1					0.158

*n*: frequency; %: percentage; SD: standard deviation; *P*: level of significance; data are shown as the mean ± 1 SD.

**Table 2:** Relationship between health statuses and demographic characteristics of the participants.

Characteristic	Physical health∗		Social health∗∗		Mental health∗∗∗	
	χ^2^	P	χ^2^	P	χ^2^	P
Age						
≤34	**29.038**	**<0.001**	3.196	0.061	**7.034**	**0.008**
≥35						
Gender						
Female	1.875	0.171	0.280	0.597	0.474	0.491
Male						
Level of education						
Elementary school & lower	**4.996**	**0.025** ∗	**4.565**	**0.033**	**4.277**	**0.039**
Secondary school & higher						
Civil status						
Married	0.256	0.613	0.648	0.421	0.000	0.983
Single						
Number of siblings						
2 & less	**13.577**	**<0.001**	0.867	0.352	**14.343**	**<0.001**
3 & more						
Monthly income						
≤500 TRY	0.029	0.865	0.002	0.967	0.227	0.634
≥501 TRY						
Number of rooms						
≤2	0.788	0.375	3.371	0.066	**5.142**	**0.023**
≥3						

Physical health	—	—	**5.173**	**0.016**	**4.853**	**0.024**
Social health		—	—	—	0.329	0.463

*Individuals with no physical health problems or with only temporary health problems that did not affect their daily lives were considered to be healthy. ∗∗Individuals who felt secure in their villages, who had a sense of belonging, who thought that everyone in their village cooperated with one another, and who believed that they could live in the village even if they did not work were considered to be socially healthy. ∗∗∗Mental health was evaluated using the BDS.
